# Mortality trends involving chronic kidney disease and electrolyte and acid–base disorders in the United States, 1999–2023: a CDC WONDER analysis

**DOI:** 10.1097/MS9.0000000000005439

**Published:** 2026-07-29

**Authors:** Zemichael Getu Alemayehu, Abenezer Shiferaw Keraga, Fikir Kibret, Yeabsra Tewdrose Tsegaye, Beamlak Getachew Woldeselassie, Bona Degefu Zerfu, Yonatan Abbawa Zewdie, Yared Mezemir Tiruneh, Yonathan Belay Kebede, Brook Lelisa Sime, Frealem Wechefo Amenu

**Affiliations:** a Addis Ababa University, College of Health Sciences, School of Medicine, Addis Ababa, Ethiopia; b Internal Medicine, Cook County Health, Chicago, USA; c Internal Medicine, Eka Kotebe Hospital & Neuroscience Institute, Addis Ababa, Ethiopia; d School of Medicine, Arba Minch University, College of Medicine and Health Sciences, Arba Minch, Ethiopia; e School of Medicine, Santé Medical College, Addis Ababa, Ethiopia

**Keywords:** CDC WONDER, chronic kidney disease, electrolyte and acid–base disorders, health disparities, mortality trends

## Abstract

**Background:**

Chronic kidney disease (CKD) is a growing global health burden, often complicated by electrolyte and acid–base disorders that exacerbate morbidity and mortality. Despite recognition of CKD-related cardiovascular outcomes, mortality patterns involving metabolic complications remain underexplored.

**Methods:**

Using CDC WONDER Multiple Cause-of-Death data (1999–2023), we identified adult deaths (≥25 years) involving CKD (ICD-10 code N18) and electrolyte and acid–base disorders (ICD-10 codes E87.0–E87.8). Age-adjusted mortality rates (AAMRs) were calculated per 100 000 population, stratified by sex, race/ethnicity, census region, and urbanization. Temporal trends were assessed using Joinpoint regression.

**Results:**

Between 1999 and 2023, 75 149 deaths occurred among individuals with both CKD and electrolyte/acid–base disorders (overall AAMR, 1.35). Mortality remained relatively stable, with a non-significant downward trend until 2008, and then rose sharply (APC, 6.54%; 95% CI, 5.33–7.77), peaking during the COVID-19 pandemic. Men had consistently higher AAMRs than women (1.63 vs 1.14). Non-Hispanic Black individuals had the highest burden (AAMR, 3.01), nearly threefold higher than that of non-Hispanic Whites. Geographic disparities were also evident, with the South and rural counties experiencing higher and progressively increasing mortality rates.

**Conclusions:**

Mortality co-involving CKD and electrolyte and acid–base disorders has more than doubled in the U.S. since 2008, with widening disparities by sex, race/ethnicity, and geography. These findings highlight the growing contribution of metabolic complications to CKD-related mortality and underscore the need for earlier detection and management of electrolyte and acid–base disturbances alongside equity-focused interventions for high-risk populations.

## Introduction

Chronic kidney disease (CKD) is a major societal health problem on a global scale. In the United States, more than 1 in 10 adults, approximately 14% of the adult population, are estimated to have chronic kidney disease. Globally, the age-standardized prevalence of CKD in adults was estimated at 14.2% in 2023. Worryingly, CKD remains largely asymptomatic in its early stages, and the majority of affected individuals are unaware of their diagnoses^[^1–3^]^. As CKD advances through its stages, the kidney’s ability to regulate electrolyte excretion becomes progressively impaired; fractional excretion of electrolytes, including sodium, potassium, and magnesium, increases steadily with declining glomerular filtration rate, a pattern further amplified in patients with comorbid diabetes mellitus^[^4^]^.

HIGHLIGHTS

- National mortality associated with chronic kidney disease (CKD) and electrolyte and acid–base disorders increased steadily in the United States from 1999 to 2023.
- Age-adjusted mortality rates more than doubled over the study period, with a marked increase in the trend after 2008.
- Significant disparities were observed by sex, race/ethnicity, geographic region, and urban–rural residence.
- These findings underscore the growing clinical and public health burden of metabolic complications in CKD and the need for improved prevention, early detection, and equitable management strategies.

The prevalence and mortality due to CKD have increased over the years. This is associated with aging populations and the increased prevalence of diabetes, high blood pressure, and obesity. Between 1990 and 2017, the number of CKD deaths worldwide rose by over 40%, and disability-adjusted life-years surpassed 35 million in 2017^[^5^]^. From 2002 to 2016, CKD disability-adjusted life-years in the United States increased by 52.6%, while CKD-related deaths increased by 58.3%. This increase includes significant regional variation, particularly in the Southern states, where socioeconomic factors contribute. Modifiable metabolic and lifestyle factors contribute substantially to diabetes, which is the leading cause of CKD-related disability and mortality. Although the age-standardized prevalence of CKD has shown signs of stabilization in recent years, the absolute burden remains high and continues to grow, driven by rising rates of diabetes, obesity, and metabolic syndrome^[^6^]^.

In the United States, non-Hispanic Black populations and individuals of lower socioeconomic status are among the demographic groups most disproportionately affected by CKD. Disparities in CKD burden and access to care persist among younger patients and individuals from minority backgrounds^[^7,8^]^. There are also distinct sex differences: women show higher rates of incidence and prevalence, while men have higher mortality rates from CKD^[^9^]^. It has been estimated that the prevalence of CKD will continue to increase in the next few decades. This shows the urgent need to engage in prevention efforts that should target modifiable risk factors, such as metabolic health, and increase awareness of the disease to limit progression and complications^[^10,11^]^.

CKD disrupts the body’s balance and may result in cardiovascular disease, stroke, and metabolic complications. These interactions contribute to cardiovascular-kidney-metabolic (CKM) syndrome, in which CKD, cardiovascular disease, obesity, and type 2 diabetes interact bidirectionally. This complex relationship is driven by multiple factors, including hyperglycemia, insulin resistance, and chronic inflammation. These processes result in diseases such as diabetic heart disease and renal fibrosis. Awareness of CKM syndrome is essential for its early identification through screening and holistic care that takes into account both biological and social health considerations. Targeted metabolic and kidney-specific therapies show promise in reducing cardiovascular and kidney complications^[^12–14^]^.

In the United States, ischemic heart disease is a major contributor to mortality trends among patients with coexisting chronic kidney disease. A comprehensive 25-year review of national mortality data from 1999 through 2023 demonstrates a significant overall decline in age-adjusted mortality rates for coexisting chronic kidney disease and ischemic heart disease, though complex disparities in the dual disease burden persist across distinct racial, gender, and geographic subgroups^[^15^]^. Electrolyte and acid**–**base disturbances (e.g., hyperkalemia and metabolic acidosis) are common complications of CKD. These conditions can lead to cardiac arrhythmias, sudden cardiac death, and worsening kidney dysfunction, all of which influence mortality trends^[^16,17^]^. While patterns in CKD-related deaths alongside major cardiovascular conditions have been described, national trends in the co-occurrence of chronic kidney disease with individual electrolyte and acid–base disorders have not been thoroughly explored.

To address these gaps, this study examines mortality trends related to CKD and electrolyte and acid**–**base disorders in the United States of America between 1999 and 2023. It pays attention to age-adjusted rates, substantial variations over time, and disparities by sex, race/ethnicity, place, and urbanization. Ultimately, this study builds on the analytical frameworks developed in previous foundational reports using the CDC WONDER database to describe an understudied group of metabolic complications and to guide targeted prevention, clinical management, and policy interventions for high-risk populations.

## Methods

### Study setting and population

Mortality data related to CKD and electrolyte and acid–base disorders in the United States were obtained from the Centers for Disease Control and Prevention Wide-ranging Online Data for Epidemiologic Research (CDC WONDER) database. CDC WONDER is a comprehensive repository of death certificate data from all fifty U.S. states and the District of Columbia. We used the Multiple Cause-of-Death Public Use Files to identify deaths in which CKD and electrolyte and acid–base disorders were recorded as either the underlying or contributing cause of death^[^18^]^. The database query was executed on 24 January 2026, and, in accordance with CDC WONDER data protection policies, cells with death counts fewer than 10 were suppressed to maintain confidentiality.

CKD was identified using the International Classification of Diseases, Tenth Revision (ICD-10) code N18 (chronic kidney disease), while electrolyte and acid–base disorders were defined using ICD-10 codes E87.0 (hyperosmolality and hypernatremia), E87.1 (hypoosmolality and hyponatremia), E87.2 (acidosis), E87.3 (alkalosis), E87.4 (mixed disorders of acid**–**base balance), E87.5 (hyperkalemia), E87.6 (hypokalemia), E87.7 (fluid overload), and E87.8 (other). These codes have been widely used in prior epidemiologic studies examining mortality related to CKD and electrolyte and acid–base disorders. Analyses were restricted to adults aged 25 years or older, as CKD-related deaths co-occurring with electrolyte and acid–base disorders are exceedingly rare below this threshold and frequently yield suppressed or unreliable age-adjusted estimates in CDC WONDER. This age cutoff also ensures comparability with prior CDC WONDER analyses of CKD-related mortality^[^19,20^]^.

Because the CDC WONDER data are publicly available and fully de-identified, institutional review board approval was not required. This study was conducted in accordance with the Strengthening the Reporting of Observational Studies in Epidemiology (STROBE) guidelines^[^21^]^.

### Data abstraction

We extracted annual data on CKD- and electrolyte- and acid–base disorder–related mortality counts, along with corresponding population estimates and demographic characteristics, including sex and race/ethnicity, for the period 1999–2023. Race and ethnicity were categorized as non-Hispanic (NH) White, NH Black or African American, NH American Indian or Alaska Native, NH Asian or Pacific Islander, and Hispanic or Latino. To maintain consistency in race/ethnicity classifications across the study period, race/ethnicity-specific analyses were restricted to years with stable CDC WONDER definitions and reliable age-adjusted mortality estimates.

CDC WONDER flagged age-adjusted mortality rates for NH American Indian or Alaska Native populations in 1999–2003, 2005, and 2007–2010 as unreliable because of small death counts; therefore, analyses for this group were restricted to the remaining years.

Mortality trends were further evaluated across U.S. states, Census regions (Northeast, Midwest, South, and West), and urban–rural residence at the county level. Counties were classified as urban or rural according to the 2013 National Center for Health Statistics Urban–Rural Classification Scheme, with large central metro, large fringe metro, medium metro, and small metro areas classified as urban and micropolitan and noncore areas classified as rural^[^22^]^. However, due to CDC WONDER system constraints, age-adjusted rates are not available for years after 2020 when county-level variables, including urbanization categories, are selected. As a result, analyses stratified by urbanization were limited to the period 1999–2020.

### Statistical analysis

Age-adjusted mortality rates (AAMRs) per 100 000 population were calculated annually using direct age standardization to the 2000 U.S. standard population^[^23^]^.

The Joinpoint Regression Program (version 5.4.0; National Cancer Institute) was used to determine temporal trends in AAMRs^[^24^]^. This approach fits log-linear regression models that test whether changes in mortality trends over time are statistically significant. In this method, log-linear regression models were fitted to evaluate annual percentage change (APC) and its corresponding 95% confidence interval (CI). A trend was classified as increasing or decreasing if the slope significantly differed from zero, with statistical significance set at *P* ≤ 0.05 using a two-tailed *t*-test.

## Results

### Overall trends

**Figure 1.**
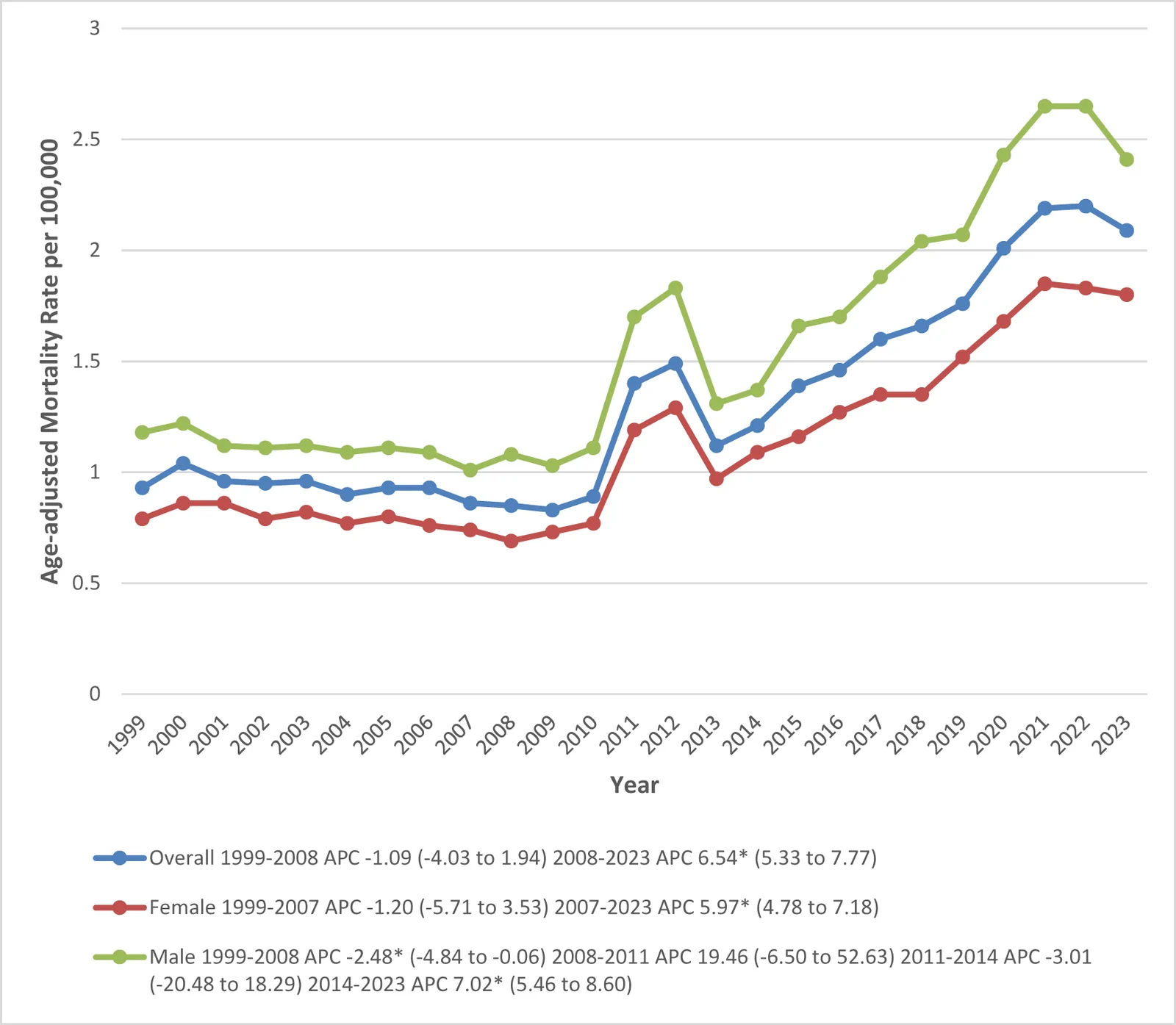
Overall and sex-stratified age-adjusted mortality rates (per 100 000) for deaths co-involving chronic kidney disease and electrolyte and acid–base disorders, United States, 1999–2023.

Between 1999 and 2023, a total of 75 149 deaths in the United States were documented with both CKD and electrolyte and acid–base disorders, corresponding to an overall age-adjusted mortality rate (AAMR) of 1.35 per 100 000 population (95% CI: 1.34–1.36) (Table 1). The annual deaths increased over time, rising from 1670 in 1999 to a peak of 5873 in 2022, followed by a slight decline to 5677 in 2023 (Supplemental Digital Content Table 1, available at: https://links.lww.com/MS9/B321, Fig. 1).

**Table 1:** Demographic characteristics and age-adjusted mortality rates for deaths co-involving chronic kidney disease and electrolyte and acid–base disorders in the USA from 1999 to 2023.

Variable	Chronic kidney disease and electrolyte and acid–base disorders–related deaths	AAMRs (95% CI) per 100 000
Overall population	75 149 (100%)	1.35 (1.34-1.36)
Sex		
Male	38 716 (51.5%)	1.63 (1.62-1.64)
Female	36 433 (48.5%)	1.14 (1.13-1.16)
Race/Ethnicity		
NH White	54 809 (72.9%)	1.12 (1.11-1.13)
NH Black or African American	16 727 (22.3%)	3.01 (2.96-3.06)
NH Asian or Pacific Islander	2661 (3.5%)	1.16 (1.11-1.21)
NH American Indian or Alaska Native	832 (1.1%)	1.91 (1.77-2.05)
Hispanic or Latino	7574 (10.1%)	1.64 (1.6-1.68)
Census region		
Northeast	13 233 (17.6%)	1.23 (1.21-1.25)
Midwest	15 683 (20.9%)	1.25 (1.23-1.27)
South	30 228 (40.2%)	1.48 (1.47-1.49)
West	16 005 (21.3%)	1.32 (1.3-1.34)
Urbanization (1999–2020 only)^a^		
Metropolitan	46 198	1.18 (1.17-1.19)
Nonmetropolitan	11 704	1.41 (1.38-1.44)

AAMR, age adjusted mortality rate per 100 000; CI, confidence interval; NH, non-Hispanic.

^a^ Urbanization data were limited to 1999–2020 owing to CDC WONDER system constraints.

Joinpoint regression identified a change in mortality trends around 2008. Between 1999 and 2008, AAMRs declined modestly from 0.93 to 0.85 per 100 000, though this trend was not statistically significant (APC, −1.09%; 95% CI, −4.03 to 1.94) (Supplemental Digital Content Table 3, available at: https://links.lww.com/MS9/B321). In contrast, from 2008 through 2023, mortality increased significantly at an average APC of 6.54% (95% CI, 5.33 to 7.77). During this interval, the AAMR more than doubled, rising from 0.85 per 100 000 in 2008 to 2.20 per 100 000 in 2022 before a modest decline to 2.09 per 100 000 in 2023 (Supplemental Digital Content Table 2, available at: https://links.lww.com/MS9/B321, Fig. 1). Notably, the sharp increases observed in 2020 and 2021 coincided with the COVID-19 pandemic, suggesting that the surge in mortality during these years may reflect pandemic-related effects on patients with CKD and electrolyte and acid–base disorders.

### Sex-stratified trends

Sex-stratified analyses identified 38 716 deaths (51.5%) among men and 36 433 deaths (48.5%) among women between 1999 and 2023 (Table 1). Over the entire study period, the overall AAMR was higher in men than in women (1.63 vs 1.14 per 100 000 population, respectively).

From 1999 to 2008, mortality rates declined modestly in both sexes, although not significantly among women. Among men, the AAMR decreased from 1.18 per 100 000 (95% CI, 1.10–1.27) in 1999 to 1.08 per 100 000 (95% CI, 1.01–1.15) in 2008, corresponding to a significant downward trend (APC, −2.48%; 95% CI, − 4.84 to −0.06). Among women, the AAMR declined from 0.79 per 100 000 (95% CI, 0.74–0.85) to 0.69 per 100 000 (95% CI, 0.65–0.74) over the same period; this trend was not statistically significant (APC, −1.20%; 95% CI, −5.71 to 3.53) (Supplemental Digital Content Table 2, available at: https://links.lww.com/MS9/B321, Fig. 1).

Beginning in the late 2000s and early 2010s, mortality increased in both sexes. Among men, the AAMR rose from 1.11 per 100 000 in 2010 to 1.70 per 100 000 in 2011 and to 1.83 per 100 000 in 2012, followed by transient fluctuations. From 2014 through 2023, male mortality increased significantly (APC, 7.02%; 95% CI, 5.46–8.60). The male AAMR reached 2.07 per 100 000 in 2019, increased to 2.43 per 100 000 in 2020, peaked at 2.65 per 100 000 in 2021 and 2022, and declined to 2.41 per 100 000 in 2023 (Supplemental Digital Content, Table 2, available at: https://links.lww.com/MS9/B321).

Among women, mortality rates increased after 2007, with a significant APC of 5.97% (95% CI, 4.78–7.18) from 2007 through 2023. Female AAMRs increased from 0.74 per 100 000 in 2007 to 1.19 per 100 000 in 2011 and 1.29 per 100 000 in 2012, followed by transient fluctuations. Rates continued to rise to 1.85 per 100 000 in 2021, remaining at similarly elevated levels in 2022 (1.83 per 100 000) and 2023 (1.80 per 100 000) (Supplemental Digital Content Table 2, available at: https://links.lww.com/MS9/B321, Fig. 1).

Across all study years, AAMRs were consistently higher among men than among women. The male-to-female AAMR ratio was approximately 1.5 during the early study period and ranged from 1.3 to 1.4 in more recent years.

### Race- and ethnicity-stratified mortality trends

Race- and ethnicity–stratified analyses demonstrated marked heterogeneity in mortality burden (Table 1). During 1999–2023, non-Hispanic (NH) White individuals accounted for 54 809 deaths (72.9%), NH Black or African American individuals for 16 727 deaths (22.3%), Hispanic or Latino individuals for 7574 deaths (10.1%), NH Asian or Pacific Islander individuals for 2661 deaths (3.5%), and NH American Indian or Alaska Native (AI/AN) individuals for 832 deaths (1.1%) (Table 1).

Over the full study period, the highest overall AAMR was observed among NH Black individuals (3.01 per 100 000; 95% CI, 2.96–3.06), followed by NH AI/AN individuals (1.91; 95% CI, 1.77–2.05), Hispanic individuals (1.64; 95% CI, 1.60–1.68), NH Asian or Pacific Islander individuals (1.16; 95% CI, 1.11–1.21), and NH White individuals (1.12; 95% CI, 1.11–1.13) (Table 1).

**Figure 2.**
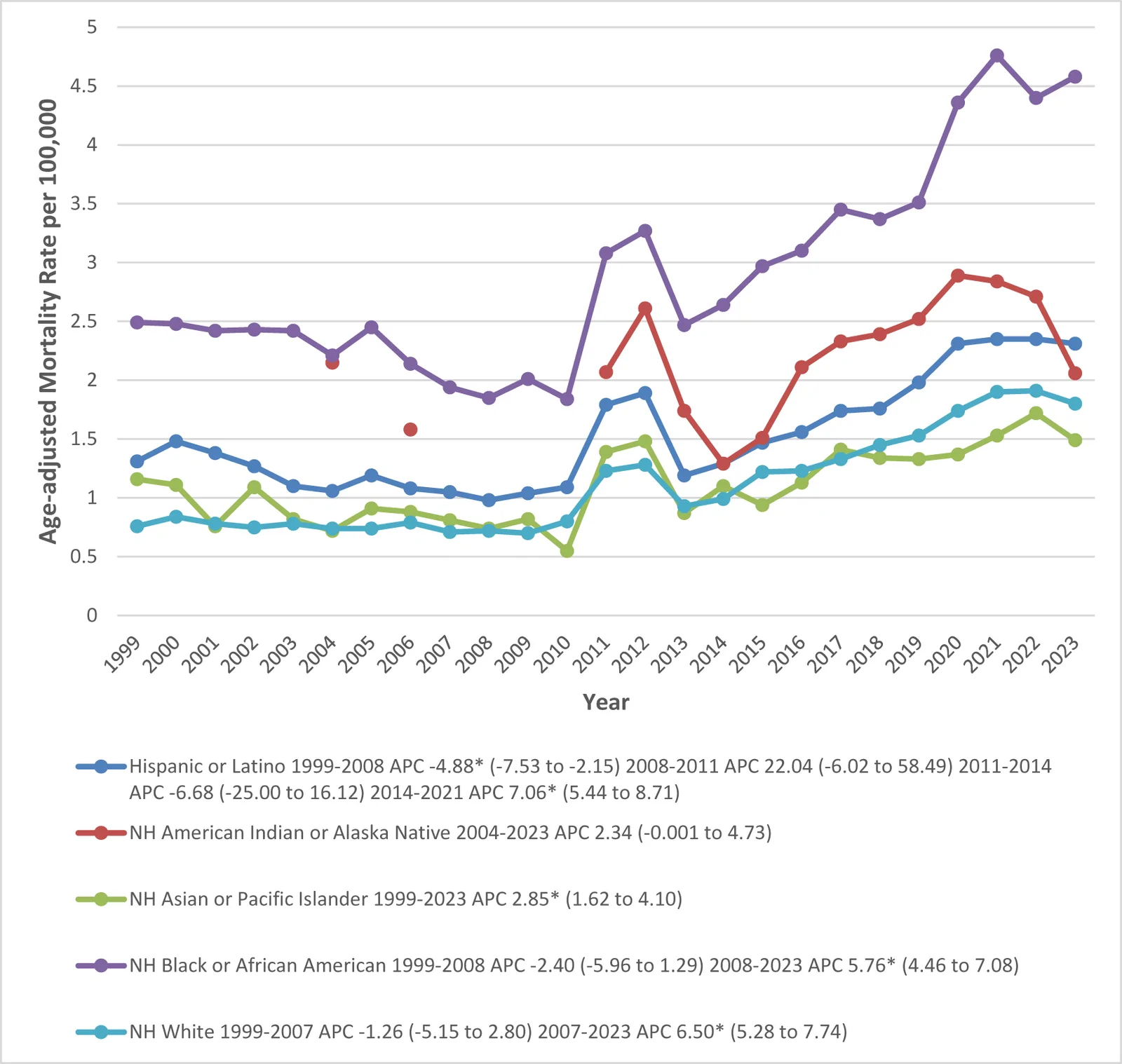
Race/ethnicity-stratified age-adjusted mortality rates (per 100 000) for deaths co-involving chronic kidney disease and electrolyte and acid–base disorders, United States, 1999–2023. Rates flagged as unreliable by CDC WONDER were excluded.

From 1999 through approximately 2007–2008, mortality rates were stable or declined modestly in most racial and ethnic groups. Among NH White individuals, AAMRs decreased from 0.76 per 100 000 in 1999 to 0.71 per 100 000 in 2007. Among NH Black individuals, rates declined from 2.49 per 100 000 in 1999 to 1.85 per 100 000 in 2008. Hispanic individuals experienced a significant decline during this period (APC, −4.88%; 95% CI, −7.53 to −2.15). Mortality rates among NH Asian or Pacific Islander individuals were relatively stable in the early study years, whereas early estimates among NH AI/AN individuals were limited by small numbers (Supplemental Digital Content Table 4, available at: https://links.lww.com/MS9/B321; Fig. 2).

Beginning in the late 2000s and early 2010s, mortality increased across nearly all racial and ethnic groups. Among NH White individuals, despite interim fluctuations, AAMRs increased significantly from 2007 onward (APC, 6.50%; 95% CI, 5.28–7.74), rising from 0.71 per 100 000 in 2007 to 1.23 in 2011, 1.91 in 2022, and 1.80 in 2023. Among NH Black individuals, rates increased significantly after 2008 (APC, 5.76%; 95% CI, 4.46–7.08), rising from 1.85 per 100 000 in 2008 to 4.36 in 2020 and 4.58 in 2023. Hispanic individuals demonstrated a significant increase after 2014 (APC, 7.06%; 95% CI, 5.44–8.71), with AAMRs rising from 1.29 per 100 000 in 2014 to 2.31 in 2020 and remaining at 2.31 in 2023.

Among NH Asian or Pacific Islander individuals, mortality increased steadily over the study period (APC, 2.85%; 95% CI, 1.62–4.10), with AAMRs rising from 1.16 per 100 000 in 1999 to 1.72 in 2022, followed by a decrease to 1.49 in 2023. Among NH AI/AN individuals, AAMRs increased from 2.07 per 100 000 in 2011 to 2.89 in 2020 before declining to 2.06 in 2023; the long-term trend from 2004 through 2023 did not reach statistical significance (APC, 2.34%; 95% CI, − 0.001 to 4.73) (Supplemental Digital Content, Table 4, available at: https://links.lww.com/MS9/B321; Fig. 2).

### Census region–stratified mortality trends

Census region–stratified analyses identified regional variation in mortality: during 1999–2023, 30 228 deaths (40.2%) occurred in the South, 16 005 (21.3%) in the West, 15 683 (20.9%) in the Midwest, and 13 233 (17.6%) in the Northeast (Table 1). Over the full study period, the highest overall AAMR was observed in the South (1.48 per 100 000; 95% CI, 1.47–1.49), followed by the West (1.32; 95% CI, 1.30–1.34), Midwest (1.25; 95% CI, 1.23–1.27), and Northeast (1.23; 95% CI, 1.21–1.25) (Table 1).

**Figure 3.**
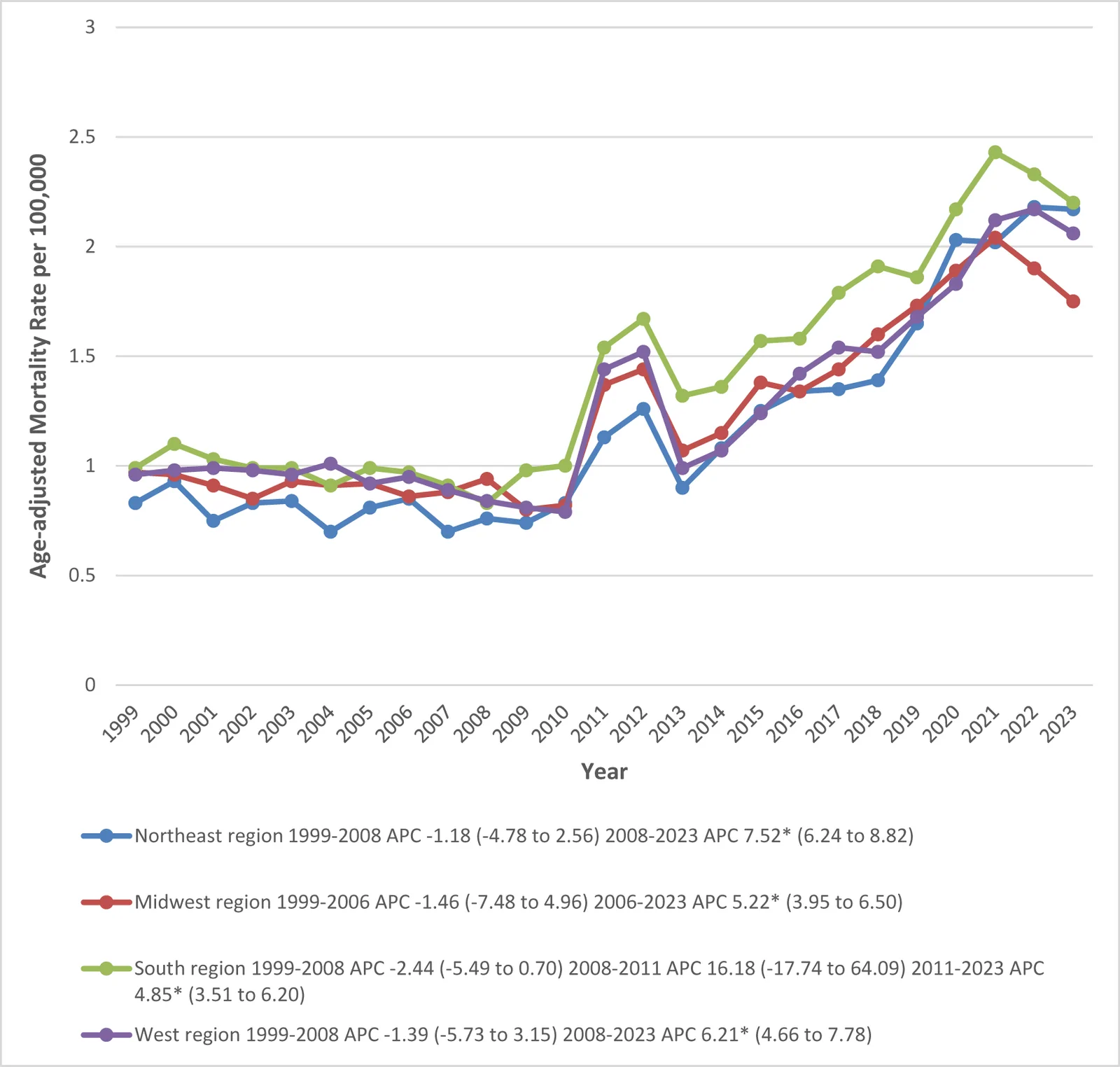
Census region–stratified age-adjusted mortality rates (per 100 000) for deaths co-involving chronic kidney disease and electrolyte and acid–base disorders, United States, 1999–2023.

From 1999 through 2006–2008, AAMRs were stable or declined modestly across regions. In the Northeast, rates decreased from 0.83 per 100 000 in 1999 to 0.70 in 2007 (Supplemental Digital Content Table 5, available at: https://links.lww.com/MS9/B321). In the Midwest, AAMRs declined from 0.97 in 1999 to 0.86 in 2006. In the South, rates decreased from 0.99 in 1999 to 0.83 in 2008, while in the West, rates declined from 0.96 in 1999 to 0.84 in 2008. Joinpoint analyses indicated that declines during this early interval were not statistically significant in most regions (Supplemental Digital Content Table 5, available at: https://links.lww.com/MS9/B321; Fig. 3).

Beginning in the late 2000s, mortality increased across all regions (Fig. 3, Supplemental Digital Content Tables 3 and 5, available at: https://links.lww.com/MS9/B321). In the Northeast, the AAMR rose from 0.76 per 100 000 in 2008 to 1.13 in 2011 and 1.26 in 2012, followed by continued increases to 2.03 in 2020 and 2.18 in 2022; the rate was 2.17 in 2023 (APC 2008–2023: 7.52%; 95% CI, 6.24–8.82). In the Midwest, the AAMR increased from 0.94 per 100 000 in 2008 to 1.37 in 2011 and 1.44 in 2012, rising further to 1.73 in 2019 and 2.04 in 2021 before declining to 1.75 in 2023 (APC 2006–2023: 5.22%; 95% CI, 3.95–6.50).

In the South, rates increased from 0.83 per 100 000 in 2008 to 1.54 in 2011 and 1.67 in 2012, with sustained elevations thereafter, reaching 2.17 in 2020, 2.43 in 2021, and 2.20 in 2023 (APC 2011–2023: 4.85%; 95% CI, 3.51–6.20). In the West, AAMRs increased from 0.84 per 100 000 in 2008 to 1.44 in 2011 and 1.52 in 2012, rising to 1.83 in 2020 and 2.17 in 2022, with a value of 2.06 in 2023 (APC 2008–2023: 6.21%, 95% CI, 4.66–7.78) (Supplemental Digital Content Table 5, available at: https://links.lww.com/MS9/B321).

### Urbanization-stratified mortality trends

Urbanization-stratified analyses for the period 1999–2020 identified 46 198 deaths in metropolitan counties and 11 704 deaths in nonmetropolitan counties. These counts reflect only deaths with available urbanization classification during 1999–2020 and therefore are not directly comparable to the full 1999–2023 cohort totals (Table 1). Over the study period, the overall AAMR was higher in nonmetropolitan than in metropolitan areas [1.41 per 100 000 (95% CI, 1.38–1.44) vs 1.18 per 100 000 (95% CI, 1.17–1.19), respectively] (Table 1).

From 1999 to 2008, AAMRs declined modestly in metropolitan areas, decreasing from 0.94 per 100 000 (95% CI, 0.89–0.99) in 1999 to 0.79 per 100 000 (95% CI, 0.74–0.83) in 2008. During the same interval, mortality rates in nonmetropolitan areas remained higher and showed less consistent change, measuring 0.97 per 100 000 (95% CI, 0.87–1.08) in 1999 and 1.13 per 100 000 (95% CI, 1.01–1.24) in 2008. Joinpoint analysis indicated a nonsignificant decline in metropolitan counties during 1999–2008 (APC, −1.48%; 95% CI, −5.16 to 2.35), whereas nonmetropolitan counties demonstrated a significant overall increase during 1999–2020 (APC, 4.71%; 95% CI, 3.68–5.74) (Supplemental Digital Content, Table 3, available at: https://links.lww.com/MS9/B321).

**Figure 4.**
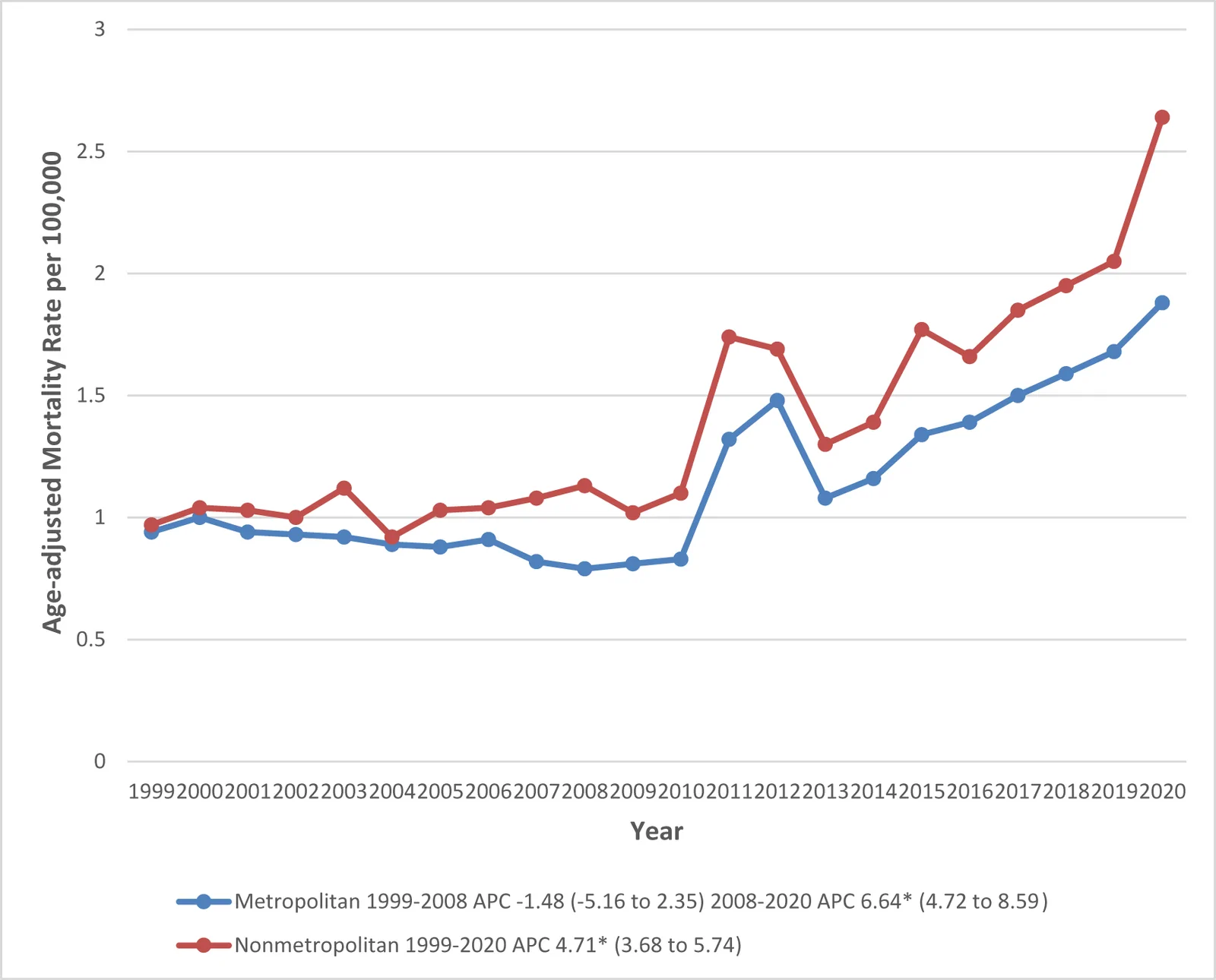
Urbanization-stratified age-adjusted mortality rates (per 100 000) for deaths co-involving chronic kidney disease and electrolyte and acid–base disorders, United States, 1999–2020.

Beginning in the early 2010s, mortality increased in both metropolitan and nonmetropolitan settings. In metropolitan areas, AAMRs rose from 0.83 per 100 000 in 2010 to 1.32 in 2011 and 1.48 in 2012, followed by continued increases to 1.68 in 2019 and 1.88 in 2020. From 2008 through 2020, metropolitan counties experienced a significant upward trend (APC, 6.64%; 95% CI, 4.72–8.59). In nonmetropolitan counties, AAMRs increased from 1.10 per 100 000 in 2010 to 1.74 in 2011 and 1.69 in 2012, rising further to 2.05 in 2019 and 2.64 in 2020, representing the highest values observed. Nonmetropolitan counties had higher AAMRs than metropolitan counties in every year, with the absolute differences growing after 2010 [Supplemental Digital Content, Table 6 (available at: https://links.lww.com/MS9/B321), Fig. 4].

## Discussion

This national analysis demonstrates that deaths involving both CKD and electrolyte and acid**–**base disorders in the United States more than doubled between 1999 and 2023, with a clear inflection from relatively stable or modestly declining rates in the early 2000s to steeply rising mortality after 2008. Over 75 000 U.S. death certificates documented this combination during the study period, and the AAMR increased at an average annual rate of 6.54% after 2008, underscoring an emerging and underrecognized contributor to CKD-related mortality.

The marked inflection in mortality trends observed around 2011 warrants careful consideration of both epidemiological and reporting factors. Notably, the approximate doubling of annual death counts between 2010 (*n* = 1910) and 2011 (*n* = 3086) occurred simultaneously across all demographic and geographic subgroups, a pattern that strongly suggests a systemic driver rather than a true population-level change. The most plausible explanation is the nationwide completion of the Electronic Death Registration System (EDRS) rollout around 2011, which substantially improved the capture of contributing, rather than solely underlying, causes of death on death certificates. Because electrolyte and acid–base disorders in the setting of CKD are frequently recorded as contributing causes, enhanced electronic capture would produce an apparent surge in co-recorded deaths without any corresponding change in true mortality. Readers should therefore interpret the magnitude of the post-2008 trend with caution, as part of the observed increase may reflect improved ascertainment. Beyond this potential reporting artifact, the rising rates may be linked to a genuine increase in the prevalence of CKD^[^25,26^]^ and the growing burden of comorbid conditions such as diabetes and hypertension^[^27^]^. The co-recording of CKD and electrolyte disorders may also reflect greater disease severity or challenges in metabolic management, though this interpretation cannot be confirmed from administrative data alone^[^16,27^]^. All explanatory factors described above should be regarded as hypotheses consistent with the observed data, not as confirmed conclusions. The further sharp increase observed in 2020–2021 coincided with the COVID-19 pandemic. Patients with CKD face an elevated risk of severe COVID-19 outcomes; however, pandemic-related disruptions to healthcare access, delays in dialysis and nephrology care, and heightened metabolic stress may also have contributed to the mortality surge. None of these mechanisms can be confirmed from death certificate data alone, and they should be treated as hypotheses for future investigation. Distinguishing the direct virological effects of COVID-19 from its indirect system-level consequences remains an important area for future research.

Men consistently experienced higher AAMRs than women, with male-to-female mortality ratios of around 1.3–1.5 throughout the study period. This pattern parallels sex differences observed in other CKD-related and cardiovascular mortality trends, where men often have higher event rates and more advanced comorbid conditions at younger ages^[^19,28,29^]^. The earlier and steeper upturn in male mortality (a significant increase from 2014 onward) compared with that in women may reflect a combination of more aggressive comorbidity profiles, differential access to or use of nephrology and preventive care, and potential sex-related differences in recognition and coding of electrolyte and acid**–**base disorders on death certificates. These are plausible hypotheses that cannot be directly tested with death certificate data.

Racial and ethnic disparities were striking and persistent^[^19,30^]^. Non-Hispanic Black individuals had the highest overall AAMR, nearly three times that of non-Hispanic White individuals, with sustained and significant increases after 2008. Hispanic and non-Hispanic American Indian/Alaska Native populations also experienced elevated mortality, while non-Hispanic Asian or Pacific Islander individuals had lower absolute rates but still showed a rising trend. These patterns mirror the well-documented excess burden of CKD incidence, progression, and kidney failure among Black, Hispanic, and AI/AN populations. Our study cannot directly measure social or genetic determinants, and none of the following factors can be confirmed as causal from administrative data alone. However, factors such as social determinants of health, structural racism, and reduced access to high-quality nephrology care may help explain the disparities observed. Among possible biological contributors, APOL1 high-risk genotypes, which are more prevalent in populations of West African ancestry and have been associated with accelerated CKD progression in prior genetic studies, may partly account for the excess burden among non-Hispanic Black individuals, though this hypothesis cannot be evaluated from administrative death records alone^[^31^]^. Similarly, disparities in access to early nephrology care, transplantation, and preventive services represent plausible but unconfirmed drivers of the observed racial and ethnic differences. In the United States, Black individuals are at higher risk of morbidity and mortality associated with CKD compared with White individuals. While only 13% of Americans are Black, 32% of cases of kidney failure occur among Black individuals ^[^32^]^.

The falls or stalls, followed by sharp rises in subsequent years, could suggest that earlier gains in risk factor control may have plateaued or may have been offset by widening socioeconomic and health system inequalities, though these remain hypotheses to be tested in studies with individual-level data. The exceptionally sharp recent rises among Hispanic people since 2014 highlight a population that could be a target for focused surveillance and intervention.

Geographic analysis revealed that the South and the West had higher mortality, with all the Census regions recording significant increases from the late 2000s onward. The highest AAMR during the entire period was also found in the South, which already has a higher proportion of CKD, diabetes, and cardiovascular disease. These regional trends may be partly related to variations in population risk factors, healthcare access, patterns of Medicaid expansion, and regional practices in CKD and emergency care, factors that cannot be directly evaluated from death certificate data but warrant targeted investigation.

Urbanization-stratified analyses further showed that nonmetropolitan (rural) counties were always more likely to experience higher mortality than metropolitan counties, and that difference increased over time. In every year between 1999 and 2020, AAMRs were higher in nonmetropolitan areas, and mortality increased substantially throughout the period. These results are consistent with larger rural-urban differences in CKD outcomes and access to emergency care, including a lower concentration of nephrologists, longer travel distances to dialysis and emergency care units, and higher rates of uninsurance^[^19,33,34^]^.

The rising mortality rates associated with CKD and its metabolic complications in this study are in line with previous epidemiologic studies that showed an increasing global CKD burden. Based on the National Health and Nutrition Examination Survey (NHANES) data from 1999 to 2018, Zhuang *et al* (2024) found increasing trends in early CKD stages (G1–G3), from 9.28% to 12.93%, while advanced stages (G4–G5) increased from 0.3% to 0.51%, with these trends driven by diabetes, hypertension, and aging^[^26^]^.

Global Burden of Disease analyses (1990–2019) confirm that males face higher CKD mortality (with female rates approximately 30% lower), despite higher female prevalence^[^35,36^]^. In addition, USRDS and NHANES reports affirm Black Americans’ excess burden, with a 3–4-fold higher risk of kidney failure, elevated rates among Hispanic and AI/AN populations, and lower but rising trends among Asians, driven by diabetes, hypertension, APOL1 risk, SDOH, and transplant inequities^[^31,32^]^.

USRDS and CDC analyses confirm the South’s disproportionate CKD burden, driven by higher diabetes and cardiovascular disease prevalence, care access gaps, uneven Medicaid expansion, and practice variations^[^37^]^. NHANES and CDC rural health reports show that nonmetropolitan counties have consistently higher CKD/AKI mortality and that the prevalence of CKD is significantly higher in rural (29.2%) than in urban (10.8%) areas, representing a 2.7-fold difference (*P* < 0.0001)^[^34^]^.

Electrolyte and acid**–**base disorders are often preventable or treatable with timely recognition and appropriate management^[^16^]^. The rising mortality trends observed in this study are therefore particularly concerning. Notably, the excess mortality burden documented here is also seen in advanced CKD populations requiring renal replacement therapy; for example, Aimanan *et al* (2025) reported significant long-term mortality among patients undergoing dialysis via radiocephalic arteriovenous fistulas, underscoring the heightened vulnerability of this subgroup to metabolic complications^[^38^]^. The data support several implications:

1. Early CKD detection and risk factor control (especially diabetes, hypertension, and heart failure) remain critical to reducing susceptibility to life-threatening electrolyte and acid–base disturbances^[^16,39,40^]^.
2. Medication safety should be prioritized, including careful monitoring of potassium and bicarbonate in patients receiving agents that affect renal handling of electrolytes or acid–base balance^[^41^]^.
3. Equity-focused interventions are needed to address the disproportionate mortality burden among Black, Hispanic, AI/AN, Southern, rural, and nonmetropolitan populations, including improved access to nephrology care, tele nephrology for rural areas, and culturally tailored CKD education^[^30,32,42^]^.

### Limitations

There are a number of limitations that should be taken into consideration when analyzing the findings of this study.

First, the data used in the analysis are ICD-10–coded death certificate data from the CDC WONDER database, which are subject to misclassification and reporting bias. CKD and Electrolyte and Acid–Base Disorders are not validated against clinical records, laboratory measurements, or imaging studies, and the cause of death may vary depending on the certifying physician and reporting jurisdiction. As a result, the frequency of CKD and Electrolyte and Acid–Base Disorders being recorded on death certificates might be under- or overrepresented, especially among the elderly, in whom several comorbid conditions coexist.

Second, the cross-sectional nature of death certificate data precludes assessment of causal or temporal relationships between CKD and electrolyte and acid–base disorders. The database does not provide information on disease onset, severity, treatment history, or clinical management. Important clinical variables – including laboratory values, dialysis status, medication use, and adherence to treatment–are unavailable, and their absence may confound observed mortality patterns.

Third, limited longitudinal individual-level data preclude controlling for comorbidities not listed on death certificates, socioeconomic status, environmental exposures, lifestyle behaviors, and genetic predispositions that may influence CKD progression and mortality outcomes.

Finally, although a peak in mortality was observed during the COVID-19 pandemic, the database was unable to distinguish deaths directly attributable to COVID-19 from indirect effects, such as healthcare disruptions, delayed care, and worsening of chronic conditions. Therefore, findings from the pandemic period should be interpreted with caution.

## Conclusion

In this nationwide study, based on mortality data from the CDC WONDER database, mortality involving both CKD and electrolyte and acid**–**base disorders increased significantly in the United States between 1999 and 2023. The increase in AAMR has been more pronounced since 2008 and has been accompanied by persistent disparities by sex, race and ethnicity, and geographic area, with disproportionately higher mortality among males, non-Hispanic Black individuals, and residents of rural areas. The findings reflect the increasing role of metabolic complications in CKD-related mortality and the need for earlier CKD detection, better control of electrolyte and acid**–**base abnormalities, and more targeted population health programs to minimize long-standing differences in kidney disease outcomes.

## Data Availability

The data we used in this study are publicly available from the Centers for Disease Control and Prevention’s Wide-ranging Online Data for Epidemiologic Research (CDC WONDER) database. The data can be accessed at https://wonder.cdc.gov. No special permissions were required to access these data.
